# Bosentan for Treatment of Pediatric Idiopathic Pulmonary Arterial Hypertension: State-of-the-Art

**DOI:** 10.3389/fped.2019.00302

**Published:** 2019-07-23

**Authors:** Yuchen Wang, Selena Chen, Junbao Du

**Affiliations:** ^1^Department of Pediatrics, Peking University First Hospital, Beijing, China; ^2^Department of Clinical Medicine, Peking University Health Science Center, Beijing, China; ^3^Division of Biological Sciences, University of California, San Diego, La Jolla, CA, United States; ^4^Key Laboratory of Molecular Cardiovascular Sciences, Ministry of Education, Beijing, China

**Keywords:** idiopathic pulmonary hypertension, target therapy, bosentan, pediatrics, pharmacology

## Abstract

Idiopathic pulmonary arterial hypertension (IPAH) is a complex disease associated with progressive deterioration. Targeted therapy for IPAH has improved in the last several decades. However, there remain many challenges to current treatment of children with IPAH, including poor prognosis and a median survival of 0.8 years. Endothelin-1 (ET-1) appears to be a key mediator in the pathogenesis of IPAH, with elevated concentrations in the plasma. Bosentan, an endothelin receptor antagonist, has been confirmed in Food and Drug Administration (FDA) to effectively treat IPAH when administered in recent studies. This review focuses on related studies and advance of bosentan in the treatment of IPAH in children.

Pulmonary arterial hypertension (PAH) is a progressively deteriorative disease characterized by an increase of pulmonary vascular resistance (PVR) caused by the vascular structural remodeling of pulmonary arteries, likely causing right ventricular failure (1). Nowadays, the 6th WSPH Task Forces proposes (2) to include PVR equal to or over three Wood units in the definition of all forms of pre-capillary PH associated with mean pulmonary artery pressure (mPAP) > 20 mmHg (3). Idiopathic pulmonary arterial hypertension (IPAH) is one of the most common PAH categories in children (4). The damage of vascular endothelial function is one of the key factors in the pathogenesis of IPAH, although it has a complex pathogenesis (5). Increased levels of endothelin-1 (ET-1) in the plasma induce smooth muscle cell proliferation and fibrosis. The activation of endothelin receptors (ETRs) enhances the adhesion and chemotaxis of neutrophils that further aggravate vascular injury. Genetics (6), autoimmune-related pulmonary vascular injury, serotonin, and metalloproteinase damage to the vascular wall, and the inhibition of Kv channels are all involved in its pathogenesis.

In recent years, epidemiological characteristics of pediatric PH in the Netherlands and the United Kingdom showed that pediatric IPAH/HPAH accounted for 46–70 and 35–60% of PAH in children, respectively (7). In the Netherlands, the incidence of pediatric pulmonary hypertension is 3.0/1,000,000 and the prevalence rate is 20/1,000,000. In the United Kingdom and New Zealand, the incidences of IPAH are 0.48/1,000,000 and 0.7/1,000,000, and the prevalence rate is 2.1/1,000,000 and 4.4/1,000,000, respectively (8). However, 7.8% of children have a family history (UK). Without targeted treatment in children with IPAH, only 37% of patients survive within 1 year after diagnosis. According to the National Institutes of Health Registry, the median survival time of traditional treatment was only 10 months before 1995, and the 5 year survival rate was as low as 25% (9). However, with the progress of targeted therapy, IPAH treatment in children has undergone tremendous changes in the past decade (10) and has made great progress.

Meta-analysis reported that targeted therapy for PAH, including endothelin receptor antagonists, phosphodiesterase five inhibitors and prostacyclins, improved functional class, hemodynamics, and long-term prognosis in adults, but the efficacy has not yet been confirmed in children (10). In the past decade, the studies on bosentan, an endothelin receptor antagonist, for the treatment of pediatric IPAH have made new progress in several prospects (11–13).

## Pharmacology of Bosentan in Pediatric IPAH

Bosentan, the first orally active and dual antagonist for ETRs, was put onto use. By blocking the ETRs and inhibiting ET-1 function, it results in an inhibition of endothelial cell proliferation.

### Introduction of Endothelin Receptors (14, 15)

The endothelium and ETRs play an important role in regulating pulmonary vascular development. ET-1 is a crucial part of endothelin isoforms, and has been detected in all types of vessel. ET-1 is comprised by 21 amino acids that are synthesized and released from endothelial cells with a three-step process: the gene encodes prepro-ET-1 by proteolytic cleaving the initial of the signal peptidase, and the generated pro-peptide is further cleaved by the enzyme furin convertase to big-ET-1 precursors. Big-ET-1 needs endothelin-converting enzyme (ECE) to biologically activate under particular conditions and transforms to mature form. Both physical and chemical stimuli contribute to alterations in the production. After that, ET-1 is secreted by constitutive pathway to interact with ET receptors to contribute to vasomotor tone or the regulated pathway in response to external stimuli from Weibel-Palade bodies.

ET-1 interacts with ET-A receptors on the smooth muscle to mediate constriction in all arteries and veins, while ET-B exhibits vascular contractions under normal physiologic conditions by releasing vasodilators such as nitric oxide. The above mechanisms make the homeostasis physiological.

### ET-1 in IPAH (15)

In pediatric IPAH, plasma endothelin levels are elevated, and ECE activity is enhanced (16). ET-1 is highly expressed in the lung. The overproduction of ET or overactivation of ET-A receptors and reduced ET-B receptors under pathophysiological conditions will result in an intense vasoconstriction of vessels and an effect to stimulate matrix production and cell proliferation, which results in the fibrosis and inflammation of PAH. The levels of ET-1 correlated with the levels of PVR. As such, endothelin receptor antagonists likely play a role in the treatment of IPAH (17, 18).

### Mechanism for Bosentan in Treating PAH

**Figure 1:**
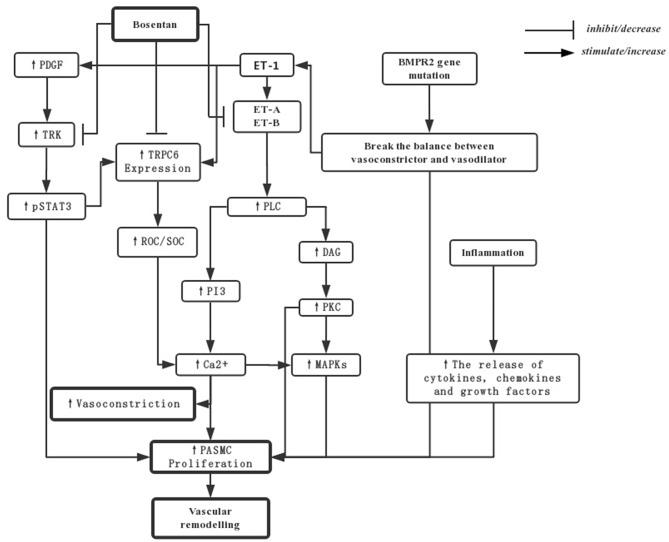
The mechanisms for bosentan in treating PAH. PDGF-mediated activation of TRKs can upregulation of TRPC6 expression by activating of STAT3 phosphorylation. The resultant increase in the activity of pSTAT3 and ROCs and SOCs would increase Ca^2+^ to stimulate PASMCs and vasoconstriction. The over-expression of ET-1 can increase levels of PVR by ET-A and ET-B activation through PLC and the downstream in PI3 and DAG pathways. What's more, the balance between vasoconstrictive and vasodilatory mechanisms may be broken by the mutations in BMPR2, and the release of inflammation factors may also induce proliferation of cells. Bosentan, as an endothelin receptor antagonist, directly downregulates TRPC6 and TRK expression by repressing the gene transcription or translation, in addition to blockade of endothelin receptors and their downstream signal transduction pathway traditionally. PASMCs, pulmonary artery smooth muscle cells; DAG, diacylglycerol; PKC, protein kinase C; ROCs, Receptor-operated Ca^2+^ channels; SOCs, store-operated Ca^2+^ channels; TKRs, PDGF-mediated activation of tyrosine kinase receptors; pSTAT3, transcription-3 phosphorylation; PDGF, platelet-derived growth factor; TRPC, transient receptor potential channels.

Pulmonary arterial hypertension (PAH) is mainly developed according to three factors: pulmonary vasoconstriction, vascular remodeling caused by vascular smooth muscle proliferation, and inflammation. The following picture shows the main mechanism of bosentan in PAH (14, 19, 20) (Figure 1).

### Pharmacokinetics of Bosentan (18, 21–24)

In adult, bosentan attains peak plasma concentrations in 3–5 h with the maximum plasma concentration (C_max_) of ~1,000 ng/ml. The terminal elimination half-life (t_1/2_) is about 5.4 h and is unchanged at steady state in healthy adult subjects with a 50% bioavailability. The steady-state concentrations are achieved within 3–5 days after multiple-dose administration. Bosentan is ~98% bound to albumin and multiple-dose administration has a volume of distribution of 30 L and a clearance of 17 L/h. Bosentan is mainly metabolized by CYP2C9 and 3A4 isoenzymes, and therefore, kidney function has a slight influence over it (18). The excretion of the metabolites via the bile constitutes the major pathway of elimination. The first-pass effect of bosentan is maximally 20% due to the clearance and the blood/plasma distribution ratio is 0.6.

The pharmacokinetics of bosentan in pediatric PAH patients and healthy adults are similar (24) according to the C_max_, T_max_, AUC, and values for t_1/2_. The activity of CYP3A4 and P2C9 surges after birth, and reaches adult levels after 1 year-old. Furthermore, the extent of the reduction in exposure to bosentan in the pediatric patients is similar with adult.

Moreover, Beghetti et al. (25) showed that the dose of bosentan from 2 to 4 mg/kg did not alter the plasma concentrations in children, and also the concentration-time of doses of 2 and 4 mg/kg overlapped, suggesting that an exposure plateau was reached at a dose of 2 mg/kg, twice a day, likely due to the smaller size of their intestinal surface area and different absorption characteristics. The apparent t_1/2_ of bosentan was similar to that in children based on the above research.

## Bosentan as a Targeted Therapy Drug for Pediatric IPAH

In European Medicines Agency (EMA), Food and Drug Administration (FDA), and China Food and Drug Administration (CFDA) highlight (26, 27), bosentan is indicated for the treatment of PAH to improve exercise capacity and symptoms in patients with WHO functional class III. The efficacy has been shown in primary PAH (idiopathic and familial), PAH secondary to scleroderma without significant interstitial pulmonary disease, congenital systemic-to-pulmonary shunts and Eisenmenger syndrome. In Europe and China, the recommended initial and maintenance dose is 2 mg/kg, although there are no data available on the safety and efficacy in pediatric cases. But bosentan has already been listed by the FDA as an indication for children at the age of 3 years old and older with IPAH or congenital PAH.

At present, numerous RCTs have shown that adult patients benefited from bosentan with respect to the six-minute walking test (6MWT), functional class (FC), safety and long-term prognosis (28–30), but the effects of bosentan on IPAH have not been completely defined in children. Due to the limitation of the investigation in child populations, there have been only a small number of cohort studies regarding the effect of bosentan in children with IPAH, although few RCT clinical studies have been conducted.

Yung et al. (31) performed a cohort study of 77 children with IPAH, indicating that the use of calcium channel blockers (CCB) in 31 children positive for acute vascular response (AVR-positive) resulted in 1, 5, and 10 year survival rates of 97, 97 and 81%, and treatment success rates of 84, 68, and 47%, respectively. Following the rise of the “New Drug Age” after 1995 (32, 33), drugs such as bosentan and sildenafil appeared on the market, yielding 1, 5, and 10 year survival rates of 97, 97, and 78%, and the treatment success rates of 93, 86, and 60%, respectively. These data support the view that new targeted drugs can improve the survival rate and quality of life of patients.

### Change in Six-minute Walking Test Before and After Treatment of Bosentan

In 2006, Maiya et al. (34) examined the short-term effect of bosentan. They observed 10 pediatric IPAH patients who were treated with bosentan for 6 months. The six-minute walking test showed that five out of the 10 children improved substantially, with a mean improvement of 176.8 m, and a mean improvement of 68 m among the entire group. Hislop et al. (16) confirmed that again in 2011, as demonstrated by a 6-minute walking distance increase from a baseline of 271 m to 370 m. To explore the long-term effects of targeted drug therapy, Raposo-Sonnenfeld et al. (35) reviewed seven patients in 2007, five of whom received sildenafil, and two received bosentan. All patients showed improvements in the 6-minute walking distance, from an average baseline of 394.2 m to 464.2 m in 6 months and 526.7 m after 2 years, demonstrating long-term period effects. Similar results were also seen in a 7 year retrospective cohort study in 2010 by Moledina et al. (36), which showed that 23 patients improved their 6-minute walking distance from 285 to 385 m after received bosentan treatment.

Additionally, bosentan was used for prostacyclin replacement therapy. Ivy et al. (37) conducted a cohort study of eight children with IPAH in 2004. Eight children received bosentan on the background of 1 year of prostacyclin treatment. At 6 months, the 6-minute walking distance increased from 498 m under the use of prostacyclin to 518 m. The 6-minute walking distance of IPAH children after bosentan treatment was increased by an average of 30–176 m in 6 months, and 100–132.5 m in 2 years. The cardiopulmonary function changed greatly in short and long periods (Table 1).

**Table 1:** Six-minute walking distance (6MWD) of pediatric IPAH patients before and after the treatment of bosentan.

**Numbers**	**F/M**	**Age, y**	**Treatment**	**6MWD before treatment**	**6MWD after treatment**	**References**
8	4/4	12.8 (8.5–17.8)	Bosentan add-on after epoprostenol for >1 year	498 m	518 m	Ivy et al. (37)
20	15/5	8.0 (1.2–17.0)	Bosentan	6WMT: 245 m (*n* = 10)	6WMD: 421.8 m (*n* = 10)	Maiya et al. (34)
7	3/4	9.6 (1.0–16.0)	Sildenafil (*n* = 5) Bosentan (*n* = 2)	394.2 m	6 mon: 464.2 m 2 y: 526.7	Raposo-Sonnenfeld et al. (35)
64	26/38	4.3 (1.5–8.9)	Bosentan (*n* = 23) Prostanoids (*n* = 15) Sildenafil (*n* = 9) Combination therapy (*n* = 11) Calcium channel antagonists (*n* = 6)	6WMT: 285 m (19 out of 23 patients taking bosentan)	6WMD: 385 m (19 out of 23 patients taking bosentan)	Moledina et al. (36)
42	26/16	9.7	Bosentan	271 m	370 m	Hislop et al. (16)

*F, Female; M, Male*.

### WHO Functional Class of Pediatric IPAH Patients Treated by Bosentan

Beghetti et al. (25) in 2009 showed that amongst 35 children treated with bosentan for 12 weeks, 29 of 35 remained (82.9%) unchanged, 5 of 35 (14.3%) upgraded one class, and one of 35 (2.9%) worsened one class. The cardiac function of patients also improved more significantly than before. Raposo-Sonnenfeld et al. (35) reported in 2007 that six of seven children with IPAH were graded III or IV prior to treatment with bosentan, whereas all seven children were graded I or II following 2 years of treatment. Similarly, Ivy et al. (38) retrospectively reviewed 36 children with IPAH treated with bosentan, of whom 14 (38%) children elevated by one functional class and 12 children remained unchanged. Berger et al. (39) reported similar proportions, in which 11 of 28 (39.3%) children improved from the baseline value, and two of 28 (7.1%) children worsened. Furthermore, Hislop et al. found that the WHO functional class changed from 2.8 to an average of 2.4 after 6 months of bosentan treatment (16).

For prostacyclin replacement, eight children with IPAH in the study by Ivy et al. (37) received prostacyclin treatment with an average functional class of 2.3 at baseline. When prostacyclin was reduced and bosentan added for 1 year, their average functional class improved to 2.0, and two out of eight cases who received prostacyclin replacement therapy increased their functional class by one grade, indicating that bosentan as a replacement therapy could increase functional class while reducing adverse effects. In 2006, Maiya et al. (34) obtained similar results.

To compare the effect of monotherapy and combined therapy, Rosenzweig et al. (40) conducted a 2-year study in which 38 patients used bosentan alone and 40 patients used prostaglandins in addition to bosentan for treatment. Overall, 36 (46%) patients improved by at least one class, 34 (44%) patients remained in the same class, and 8 (10%) patients worsened by one class. There was no statistical difference between the two groups, but the effect appeared more pronounced for bosentan alone than for combined prostaglandins.

In summary, after 6 months to 1 year of treatment with bosentan, the functional class in 20–46% patients improved, 44–55% patients remained unchanged, and a small number of patients declined due to progression of the original diseases. Bosentan demonstrated greater efficacy in treatment than prostaglandins and bosentan used in conjunction with prostaglandins (41) (Table 2).

**Table 2:** WHO functional class of pediatric IPAH patients treated by bosentan.

**Numbers**	**F/M**	**Age, y**	**Treatment**	**WHO functional class before treatment**	**WHO functional class after treatment**	**References**
8	4/4	12.8 (8.5–17.8)	Bosentan add-on after epoprostenol for >1 year.	The mean class before treatment is 2.3 WHO II: *n* = 6 WHO III: *n* = 2	The mean class after treatment is 2.0 WHO I: *n* = 2 WHO II: *n* = 4 WHO III: *n* = 2	Ivy et al. (37)
86	49/37	11.0 (0–18.0)	Bosentan concomitant Prostanoid (*n* = 42) Bosentan (*n* = 44)	WHO I: *n* = 6 WHO II: *n* = 34 WHO III: *n* = 32 WHO IV: *n* = 6	Improved one class: *n* = 36, remained stable: *n* = 34, worsened one class: *n* = 8 (78 out of 86 patients had WHO assessments)	Rosenzweig et al. (40)
20	15/5	8.0 (1.2–17.0)	Bosentan	WHO II: *n* = 1 WHO III: *n* = 11 WHO IV: *n* = 8	Improved one class: *n* = 8, remained stable: *n* = 9, worsened one class: *n* = 2	Maiya et al. (34)
7	3/4	9.6 (1.0–16.0)	Sildenafil (*n* = 5) Bosentan (*n* = 2)	WHO II: *n* = 1 WHO III: *n* = 5 WHO IV: *n* = 1	All of them are in Class I or II	Raposo-Sonnenfeld et al. (35)
36	15/21	7.0 (2.0–22.0)	Bosentan	WHO II: *n* = 23 WHO III: *n* = 13	Improved one class: *n* = 5 remained stable: *n* = 29, worsened one class: *n* = 1 WHO I: *n* = 2 WHO II: *n* = 23 WHO III: *n* = 10	Beghetti et al. (25)
64	26/38	4.3 (1.5–8.9)	Bosentan (*n* = 23) Prostanoid (*n* = 15) Sildenafil (*n* = 9) combined therapy (*n* = 11) Calcium channel antagonists (*n* = 6)	WHO II: *n* = 12 WHO III: *n* = 34 WHO IV: *n* = 18	They all get improved, and the mean class after treatment is 3.0	Moledina et al. (36)
36	16/20	10.5 (1.0–16.0)	Bosentan (*n* = 11) Bosentan concomitant prostanoid (*n* = 25)	WHO I: *n* = 3 WHO II: *n* = 12 WHO III: *n* = 16 WHO IV: *n* = 3	Improved one class: *n* = 14, remained stable: *n* = 12, worsened one class: *n* = 6 (32 out of 36 patients had assessments)	Ivy et al. (38)
42	26/16	9.7 (no data)	Bosentan	Mean class: 2.9 WHO I: *n* = 2 WHO II: *n* = 8 WHO III: *n* = 25 WHO IV: *n* = 7	The mean class is 2.4 No detailed data	Hislop et al. (16)
36	21/15	6.8 (2.0–12.0)	Bosentan	WHO II: *n* = 17 WHO III: *n* = 11	Improved one class: *n* = 11, remained stable: *n* = 15, worsened one class: *n* = 2	Berger et al. (39)

*F, Female; M, Male*.

### Hemodynamic Parameters Change After Treatment of Bosentan

Barst et al. (24) showed that 19 pediatric IPAH patients showed significant improvement in hemodynamics after being treated with bosentan for 12 weeks. Raposo-Sonnenfeld et al. (35) reached the same conclusion. Two years later, the mPAP decreased from 91.2 mmHg to 86.2 mmHg. However, in a retrospective observational study by Hislop et al. (16), the hemodynamics did not significantly change but showed a downward trend: mPAP decreased from 48.8 to 48.3 mmHg, and PVR decreased from 16.5 U.m^2^ to 14.1 U.m^2^.

Later on, to know the hemodynamic differences between bosentan monotherapy and combined prostaglandin therapy, Rosenzweig et al. (40) studied 49 patients, including 25 treated with bosentan monotherapy. They found that those treated with bosentan monotherapy had a decrease in mPAP of 9 mmHg and a decrease in PVR of 6 U.m^2^. Twenty-four patients treated with combined prostaglandin therapy showed a decrease in mPAP of 4 mmHg and a decrease in PVR of 3 U.m^2^, which indicated that monotherapy resulted in better effects than when used in combination. Furthermore, bosentan had a better therapeutic effect than prostaglandins when used alone. Moledina et al. (36) found that the PVR of the bosentan group was decreased by 23% on average, and that of the prostaglandins group decreased by 17% (Table 3).

**Table 3:** Hemodynamics at bosentan initiation and after at least 6 months of treatment.

**Numbers**	**F/M**	**Age, y**	**Treatment**	**mPAP (mmHg) PVRi (U.m^**2**^) before treatment**	**mPAP (mmHg) PVRi (U.m^**2**^) after treatment**	**Conclusion**	**References**
49	No data	11.0 (0–18.0)	Bosentan concomitant prostanoid (*n* = 24) Bosentan (*n* = 25)	mPAP: 64 PVR: 19	mPAP: 57 PVR: 15 In 25 patients taking bosentan: mPAP decreased by 9 mmHg, and PVR decreased by 6 mmHg	mPAP, PVR improved	Rosenzweig et al. (40)
20	15/5	8.0 (1.2–17.0)	Bosentan	mPAP: no data PVR: 21.7	mPAP: 61.45 PVR: 21.74	No significant change in PVR, mPAP	Maiya et al. (34)
7	3/4	9.6 (1.0–16.0)	Sildenafil (*n* = 5) Bosentan (*n* = 2)	mPAP: 91.2 PVR: no data	mPAP: 86.2 PVR: no data	mPAP improved	Raposo-Sonnenfeld et al. (35)
64	26/38	4.3 (1.5–8.9)	Bosentan (*n* = 23) Prostanoids (*n* = 15) Sildenafil (*n* = 9) Combination therapy (*n* = 11) Calcium channel antagonists (*n* = 6)	mPAP: 58 PVR: 19.7	mPAP: no data PVR: improved by 23% (23 patients taking bosentan)	PVRi improved	Moledina et al. (36)
42	26/16	9.7	Bosentan	mPAP: 48.8 PVR: 16.5	mPAP: 48.3 PVR: 14.1	No significant change in mPAP, PVRi	Hislop et al. (16)

*F, Female; M, Male*.

### Survival in Patients Treated With Bosentan (42)

Ivy et al. (38) conducted a retrospective observational study in the USA. After the treatment with bosentan, in conjunction with or independent of other PAH-specific therapies, the survival at 1-, 2-, 3-, and 4- years was 98, 88, 82, and 82%, respectively. Despite the differences in children' drug response, bosentan therapy significantly prolonged survival time. A similar conclusion was obtained in the study by Barst et al. (30), Hislop et al. (16), and Berger et al. (4). Each study showed a significant survival benefit with bosentan in pediatric IPAH patients, and survival after bosentan treatment for 3 years was around 90%.

In addition, Simpson et al. (43) compared the survival between bosentan targeted therapy and historic therapy. They reviewed IPAH children at the Royal Melbourne Children's Hospital. Seven children received bosentan treatment and 12 children received historic treatment (such as aspirin, digoxin, CCB, etc.). Survival in the bosentan-treated group was 100% at 3 years and 75% at 5 years, compared with 33% at both time-points in the historic control group. Furthermore, Rosenzweig et al. (40) compared the therapeutic efficacy of bosentan with bosentan plus prostaglandin. They noticed that the 1 and 2 year survival rate of the entire group was 100 and 88%, respectively, 98 and 94% in the bosentan-treated group, and 98 and 89% in the bosentan plus prostaglandin-treated group, respectively. Moledina et al. (36) found that the survival in bosentan monotherapy appeared greater than in combined therapy, and the survival of the entire group was around 89% in 1 year, 84% in 3 years, and 75% in 5 years.

In conclusion, the survival of each group after bosentan treatment fluctuated from 89 to 100% in 1 year, from 89 to 95% in 2 years, and from 84 to 95% in 3 years. Survival was significantly higher than seen in traditional treatments, however there was no significant difference in survival between bosentan monotherapy and combined therapy (Table 4).

**Table 4:** Patients' survival of treatment with bosentan.

**Numbers**	**F/M**	**Age, y**	**Treatment**	**Survival**	**Conclusion**	**References**
86	49/37	11.0 (0–18.0)	Bosentan concomitant Prostanoid (*n* = 42) Bosentan (*n* = 44)	The entire group: survival at 1- and 2-year was 100 and 88%, respectively Bosentan: 1- and 2-year survival was 98 and 94% Concomitant prostanoid: 1- and 2- year survival was 98 and 89%, respectively	Bosentan prolongs the life	Rosenzweig et al. (40)
7	6/1	7.4	Bosentan	The 3- and 5-year survival was 100 and 75%, respectively	Survival improved	Simpson et al. (43)
64	26/38	4.3 (1.5–8.9)	Bosentan (*n* = 23) Prostanoids (*n* = 15) Sildenafil (*n* = 9) Combined therapy (*n* = 11) Calcium channel antagonists (*n* = 6)	The entire group: 1-, 3-, and 5-year survival was 89, 84, and 75% for the entire group, respectively	The entire group improved, but with no data in bosentan, and there had no difference among bosentan, prostanoids, and sildenafil	Moledina et al. (36)
36	16/20	10.5 (1.0–16.0)	Bosentan (*n* = 11) Bosentan plus prostanoid (*n* = 25)	The entire group: survival at 1-, 2-, 3-, and 4-year was 98, 88, 82, and 82%, respectively	Most children improved in survival	Ivy et al. (38)
42	26/16	9.7	Bosentan	The survival values was 95, 95, 95, and 55% in 1, 2, 3, and 5 years, respectively	Effective in the long-term management	Hislop et al. (16)
122	73/49	15.0	53 out of 122 patients taking endothelin receptor antagonist: eight out of 53 received sitaxsentan, 45 out of 53 received bosentan	The survival of 122 patients in 6 months, 1- and 2-year was 99, 95, and 90%, respectively	Survival has been improved by targeted therapy	Barst et al. (30)
36	21/15	6.8 (2.0–12.0)	Bosentan	Estimated long-term survival at 2- and 4-year was 91.2 and 84.0%, respectively	There was an improvement in survival	Berger et al. (4)

*F, Female; M, Male*.

### Safety and Tolerability in Treatment With Bosentan (44–47)

Common adverse reactions are respiratory tract infections, pyrexia, elevations of liver aminotransferases and liver failure (26).

Barst et al. (24) conducted a cohort study of 19 children with IPAH in 2003. Flushing, headache, edema, tachycardia, tremor, and increased liver transaminase levels were reported to be the most frequent adverse events in children who were treated with bosentan. These effects were also reported in the studies of Rosenzweig et al. (40), Simpson et al. (43), Ivy et al. (38), and Beghetti et al. (48).

No patients died during Barst's observation of bosentan treatment (22). However, in Rosenzweig et al. (40), two patients died from right heart failure in the bosentan treatment group, three died in the bosentan plus prostaglandins treatment group, two patients died from hemoptysis and acute respiratory distress syndrome, and one patient died from worsening right heart failure; and all deaths were considered as due to the clinical progression of IPAH. Similar results demonstrated by Simpson et al. (43), shows that three patients needed additional intravenous prostacyclin due to the poor efficacy of bosentan. However, lung transplant was not necessary. Unlike prior studies, among the seven children treated with bosentan in Raposo-Sonnenfeld et al. (35), there were no definite side effects with the exception of menorrhagia after 1 year of treatment. The side effects mentioned above were also seen in the studies by Ivy et al. (37) and Berger et al. (39), where six people died although causes were likely unrelated to bosentan.

In summary, causes for discontinuation of bosentan treatment for pediatric IPAH include worsening heart failure, and progressive pulmonary hypertension. Bosentan-related side effects were less than those related to prostacyclin, and most patients had no serious outcomes. The majority of patients' deaths was attributed to the progression of pulmonary hypertension (Table 5).

**Table 5:** Safety and tolerability in pediatric patients treated with bosentan.

**Numbers**	**F/M**	**Age, y**	**Treatment**	**Safety and side effects**	**Death**	**References**
19	10/9	3.0–15.0	10 out of 19 received bosentan	No data	No death	Barst et al. (24)
86	49/37	11.0 (0–18.0)	Bosentan concomitant Prostanoid (*n* = 42) Bosentan (*n* = 44)	No data	Bosentan + prostanoid: 3 patients died: two hemoptysis and acute respiratory distress syndrome, one worsening right heart failure Bosentan: two died from right heart failure	Rosenzweig et al. (40)
7	6/1	7.4	Bosentan	No data	One patient died of a pulmonary hypertensive crisis	Simpson et al. (43)
7	3/4	9.6 (1.0–16.0)	Sildenafil (*n* = 5) Bosentan (*n* = 2)	No patient suffered important side effects	No data	Raposo-Sonnenfeld et al. (35)
146	71/75	2.0–11.0	59 out of 146 patients received bosentan	30.8% had at least one safety signal	About 7.5% patients died in the entire group, but not related to bosentan	Beghetti et al. (48)
36	16/20	10.5 (1.0–16.0)	Bosentan (*n* = 11) Bosentan plus prostanoid (*n* = 25)	No data	Six died among 36 patients: one for right heart failure one for sudden death one for worsening pulmonary hypertension one for hemoptysis one for pulmonary hemorrhage one for thromboembolism	Ivy et al. (38)
36	21/15	6.8 (2.0–12.0)	Bosentan	Bosentan-related AEs occurred in 15 (41.7%) patients	Six deaths occurred, but unrelated to bosentan: three from worsen of PAH and cardiac complications, one from respiratory failure following pneumonia	Berger et al. (39)

*F, Female; M, Male*.

These retrospective studies demonstrate that bosentan improves efficacy over other targeted therapies, and the effect of bosentan monotherapy has a great influence on 6MWD and WHO functional class. The above data would provide new clinical evidence in hemodynamics and long-term efficacy. The use of bosentan (49, 50) lessened the side effects in prostacyclin with improvements in functional class and hemodynamics (51) on the basis of original prostacyclin treatment (52).

## Summary

Bosentan, as an ET-1 receptor antagonist, is an effective drug for children with IPAH. Further clinical studies of multicenter RCTs are needed to clarify its efficacy and safety, and explore the effective dosage for children (53). We look forward to novel breakthroughs in targeted therapy of IPAH in children.

## Author Contributions

JD, YW, and SC make substantial contributions to conception and design. YW and SC participate in drafting the article or revising it critically for important intellectual content. JD give final approval of the version to be submitted and any revised version.

### Conflict of Interest Statement

The authors declare that the research was conducted in the absence of any commercial or financial relationships that could be construed as a potential conflict of interest.

## Abbreviations

IPAH: Idiopathic pulmonary arterial hypertension
PVR: pulmonary vascular resistance
6MWT: six-minute walking test
FC: functional class
mPAP: mean pulmonary artery pressure.
